# A power comparison of generalized additive models and the spatial scan statistic in a case-control setting

**DOI:** 10.1186/1476-072X-9-37

**Published:** 2010-07-19

**Authors:** Robin L Young, Janice Weinberg, Verónica Vieira, Al Ozonoff, Thomas F Webster

**Affiliations:** 1 Department of Biostatistics, Boston University School of Public Health, Boston, MA, USA; 2 Department of Environmental Health, Boston University School of Public Health, Boston, MA, USA

## Abstract

**Background:**

A common, important problem in spatial epidemiology is measuring and identifying variation in disease risk across a study region. In application of statistical methods, the problem has two parts. First, spatial variation in risk must be detected across the study region and, second, areas of increased or decreased risk must be correctly identified. The location of such areas may give clues to environmental sources of exposure and disease etiology. One statistical method applicable in spatial epidemiologic settings is a generalized additive model (GAM) which can be applied with a bivariate LOESS smoother to account for geographic location as a possible predictor of disease status. A natural hypothesis when applying this method is whether residential location of subjects is associated with the outcome, i.e. is the smoothing term necessary? Permutation tests are a reasonable hypothesis testing method and provide adequate power under a simple alternative hypothesis. These tests have yet to be compared to other spatial statistics.

**Results:**

This research uses simulated point data generated under three alternative hypotheses to evaluate the properties of the permutation methods and compare them to the popular spatial scan statistic in a case-control setting. Case 1 was a single circular cluster centered in a circular study region. The spatial scan statistic had the highest power though the GAM method estimates did not fall far behind. Case 2 was a single point source located at the center of a circular cluster and Case 3 was a line source at the center of the horizontal axis of a square study region. Each had linearly decreasing logodds with distance from the point. The GAM methods outperformed the scan statistic in Cases 2 and 3. Comparing sensitivity, measured as the proportion of the exposure source correctly identified as high or low risk, the GAM methods outperformed the scan statistic in all three Cases.

**Conclusions:**

The GAM permutation testing methods provide a regression-based alternative to the spatial scan statistic. Across all hypotheses examined in this research, the GAM methods had competing or greater power estimates and sensitivities exceeding that of the spatial scan statistic.

## Background

Statistical tests applied in spatial epidemiology have two primary purposes. The first is to detect spatial variation in disease risk across a study region and the second is to identify areas of increased or decreased risk [1-4]. We consider generalized additive models (GAMs) and the spatial scan statistic; two popular methods that can be used for both purposes.

GAMs are generalizations of generalized linear models that allow nonparametric functions of covariates to be modeled in an additive framework [5]. Webster et al. (2006) used GAMs in spatial settings with a bivariate locally weighted regression (LOESS) smooth [5] and performed hypothesis tests using permutation techniques to determine whether there was spatial variation in disease risk and to locate statistically significant areas of increased or decreased risk [6]. Similar methods have been applied by other authors using tests based on permutation, bootstrap, and Monte Carlo techniques [7-10].

In previous research, we evaluated four permutation tests applied with GAMs to determine type I error rates and power estimates under simple hypotheses (Young, Weinberg, Vieira, Ozonoff, Webster: The Power of Hypothesis Testing Using Generalized Additive Models with Bivariate Smoothers, submitted) [11]. The four methods differed primarily in the determination of the *span *(neighborhood) size when applying GAMs to observed and permuted datasets. For the conditional permutation test (CPT), originally proposed by Webster et al. (2006), we selected an optimal span by applying GAMs to observed data using a range of possible span sizes. Akaike's Information Criterion (AIC) was recorded for each model and the minimal model AIC corresponded to the optimal span [6,12]. The statistic of interest, the difference in deviances of models including and excluding the bivariate LOESS smoothing term, was recorded for the observed data. GAMs were then applied to permuted datasets using the optimal span selected for the observed data to produce a conditional permutation distribution of difference in deviance statistics. We determined significance through the comparison of the observed statistic to the sampling distribution generated from the repeated permutations [6]. This method had an inflated type I error rate when applied using the nominal α cutoff. For a nominal significance level of 0.05, CPT had an estimated type I error rate of 9.5% when applied with a bivariate smoothing term. When the null hypothesis was rejected for p-values less than 0.025, the observed type I error rate fell within a 95% confidence interval of 0.05, the desired significance level [11].

The second method was a fixed span permutation test (FSPT) where the span size was determined *a priori *and was held constant for observed and permuted datasets. The test was otherwise performed in the same manner as the CPT. This test had an appropriate type I error rate [11] but the required *a priori *determination of the span size was a disadvantage (Young, Weinberg, Vieira, Ozonoff, Webster: The Power of Hypothesis Testing Using Generalized Additive Models with Bivariate Smoothers, submitted). An alternative method was the fixed multiple span permutation test (FMSPT), evaluating GAM models at three or five predetermined span sizes across the range of possible spans. A permutation test was performed at each selected span with a reduced significance cutoff, empirically determined to be α/#*Spans Examined*. The Bonferroni-like adjustment produced a slightly conservative type I error rate but the FMSPT had similar power estimates when compared to the other methods (Young, Weinberg, Vieira, Ozonoff, Webster: The Power of Hypothesis Testing Using Generalized Additive Models with Bivariate Smoothers, submitted). The final permutation method was the unconditional permutation test (UPT) where we determined the optimal span size for observed and permuted datasets through minimizing the AIC statistics. This method had an appropriate type I error rate; however it was computationally intensive and had reduced power when compared to the other methods [11]. A brief description of the hypothesis testing methods is located in Table 1.

**Table 1:** Description of Hypothesis Testing Methods and Significance Cutoffs

Hypothesis Testing Method	Abbreviation	Description	Significance Cutoff
Conditional Permutation Test	CPT	Select optimal span size for observed data by minimizing AIC statistic across range of spans. Compare difference in deviance statistic to conditional permutation distribution obtained by holding span size constant.	*0.025*

Fixed Span Permutation Test	FSPT	Select span size *a priori*. Compare difference in deviance statistic to conditional permutation distribution obtained by holding span size constant.	*0.05*

Fixed Multiple Span Permutation Test	FMSPT	Select 3-5 span sizes *a priori*. For each span size, compare the difference in deviance statistic to corresponding conditional permutation distribution obtained by holding the span size constant. Reject the null hypothesis if at least one p-value falls below the significance cutoff.	$\frac{0.05}{\#\;\text{​}Span\;sizes}$

Unconditional Permutation Test	UPT	Select optimal span size for observed data as in CPT. Compare difference in deviance statistic to unconditional permutation distribution obtained by selecting optimal span size for each permuted dataset.	*0.05*

Spatial Scan Statistic	---	Detects the most likely cluster through a likelihood ratio test comparing the likelihood of cases within to outside a circular zone of interest. P-values are obtained through Monte Carlo methods	*0.05*

The spatial scan statistic, a popular method proposed by Kulldorff and Nargawalla (1995), detects the most likely cluster through comparison of likelihoods of cases falling within and outside circular zones [13]. In recent power evaluations, the scan statistic performed well with a single circular cluster [2,14,15] but underperformed with multiple and non-circular clusters [16]. When applied to case-control data, aside from stratified analyses, the scan statistic cannot be adjusted for covariates [16]. We applied the scan statistic through SaTScan, publicly available free software [17], to reflect its application in spatial statistics and spatial epidemiology.

GAMs and the scan statistic were compared in a previous study that focused on cluster detection using aggregate data. The performance of the two methods depended greatly on the shape of the cluster and with irregularly shaped clusters, GAMs outperformed the scan statistic [18]. In this study, we applied the CPT, FMSPT, and spatial scan statistic to simulated case-control point data to estimate power for global and sensitivity for local hypothesis tests. The CPT and FMSPT were selected for comparison as they are computationally efficient, had similar power estimates in a previous study (Young, Weinberg, Vieira, Ozonoff, Webster: The Power of Hypothesis Testing Using Generalized Additive Models with Bivariate Smoothers, submitted), and neither method requires *a priori *selection of a single span.

**Figure 1:**
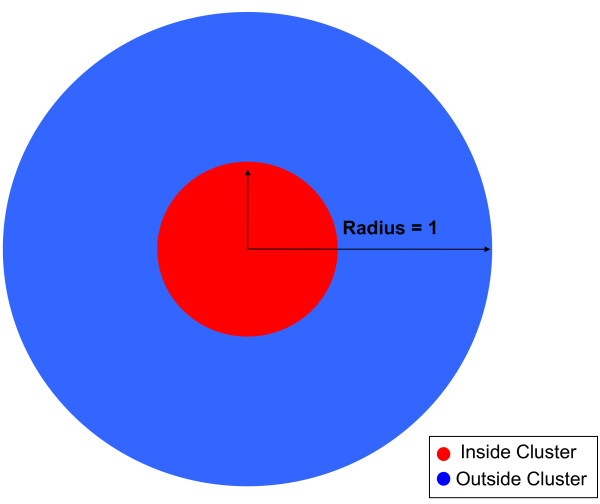
**Case 1 Study Region Diagram**. This figure is a diagram for the study region generated for Case 1.

**Figure 2:**
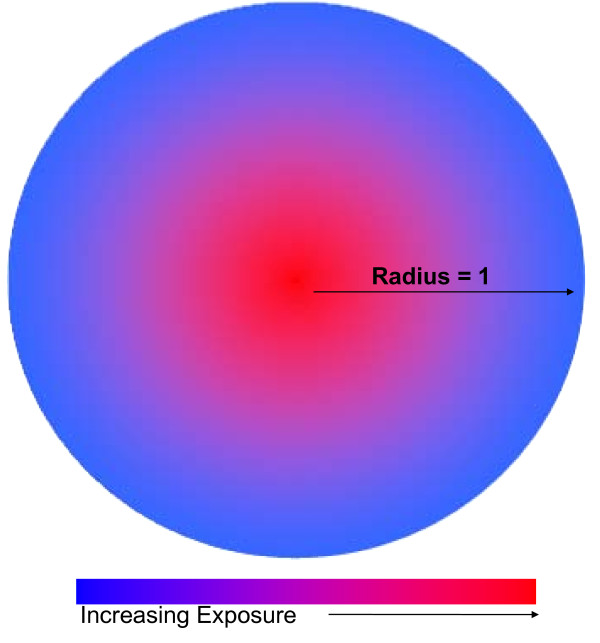
**Case 2 Study Region Diagram**. This figure is a diagram for the study region generated for Case 2.

**Figure 3:**
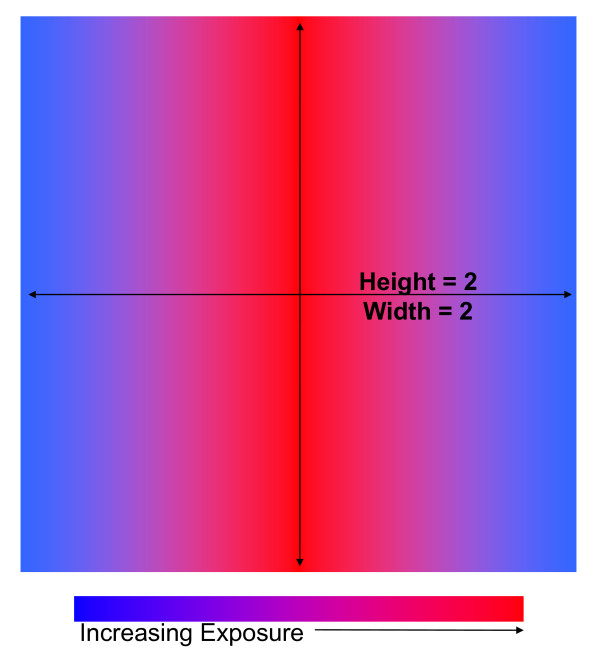
**Case 3 Study Region Diagram**. This figure is a diagram for the study region generated for Case 3.

Here, simulated data were generated in three cases: Case 1 was a circular cluster of constant increased or decreased risk centered in a circular study region (Figure 1), Case 2 was a circular study region with increased or decreased risk with proximity to the center of the region (Figure 2), and Case 3 was a square study region with increased or decreased risk with proximity to the center of the horizontal axis (Figure 3). We compared the power and sensitivity of the hypothesis testing methods for each Case.

## Methods

### Simulated Data

Simulated data had a dichotomous outcome and geographic locations generated from a bivariate uniform distribution of longitude and latitude. Odds ratios were chosen to produce a wide range of theoretical power. Odds ratios less than 1.0 indicate areas of decreased risk while odds ratios greater than 1.0 indicate areas of increased risk. For each set of parameters, 1000 datasets were simulated, each containing 1000 observations, chosen to reflect previous studies performed on the Cape Cod Family Health Study data that used GAMs as a statistical analysis technique [19-21]. Statistical analyses were applied to point data and the nominal α level was 0.05.

**Figure 4:**
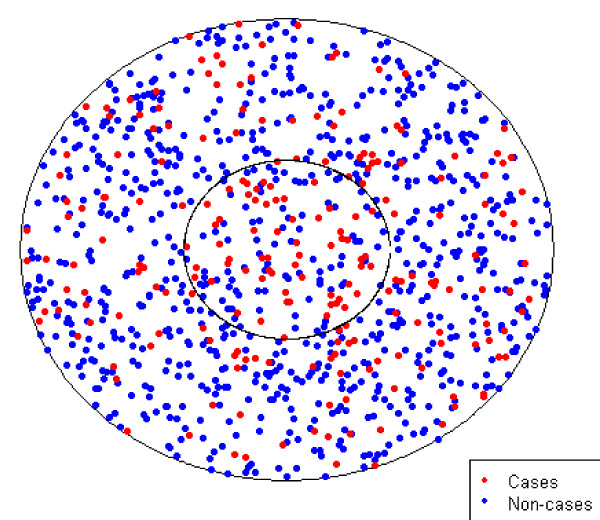
**Case 1 Example Data with Odds Ratio of 3.0**. This figure is a replicate of data simulated for Case 1 with an odds ratio and a probability of disease outside the cluster of 20%. Cases are displayed in red while non-cases are displayed in blue.

Case 1 was a circular study region that contained a circular cluster of constant risk centered in the region. (Figure 1, Figure 4) This case was a simplified version of what may be observed if subjects living within some radius of an exposure source, such as a lead smelter [22], were found to be at constant increased or decreased risk when compared to subjects living further from the source. The cluster covered 15% of the study region and approximately 150 of the 1000 subjects lived within the cluster. We considered scenarios where the probabilities of disease outside the cluster were equal to 5% or 20%. Odds ratios comparing those living within to outside the cluster were 0.5, 1.0, 1.5, 2.0, 2.5, and 3.0.

Simulated data for Case 2 reflected a circular study region with linearly increasing or decreasing logodds of disease with proximity to the center of the region. (Figure 2) This was a simplified pattern of what may be observed where increased proximity to some point, such as a lead smelter [22], increased the risk of disease. The probability of disease at the edge of the region, i.e. the subjects least exposed, was equal to 5 or 20% and odds ratios comparing subjects at the center to those at the edge of the region included 0.5, 1.0, 1.5, 2.0, 2.5, 3.0, and 3.5.

In Case 3, the study region was square with logodds of disease increasing or decreasing linearly with proximity to the center of the horizontal axis of the study region. (Figure 3) These data followed a simple pattern similar to increased risk of disease with proximity to heavy-traffic roadways [23,24]. As with Case 2, the probabilities of disease for those least exposed, i.e. living at the horizontal edges of the region, were equal to either 5 or 20%. There was no variation in disease risk across the vertical axis. The odds ratios were 0.5, 1.0, 1.5, 2.0, 2.5, 3.0, and 3.5 comparing subjects living at the center to those at the edge of the horizontal axis.

### Theoretical Power

Data for Case 1 could be appropriately analyzed using a Pearson chi-square test while data for Cases 2 and 3 could be appropriately analyzed using simple logistic regressions. We derived the theoretical power for each Case to determine how the spatial methods compare to the more simple tests. Details of these derivations are available in Additional file 1. 95% confidence intervals were computed for each power estimate. The margin of error was computed using the standard deviation of the estimated power, i.e. $95\%\;CI=\overset{\wedge }{p}\pm 1.96\sqrt{\frac{\overset{\wedge }{p}\left(1-\overset{\wedge }{p}\right)}{1000}}$, where $\overset{\wedge }{p}$ is the estimated power.

### Generalized Additive Models (GAMs)

We applied GAMs to simulated data using a bivariate LOESS smoothing term to adjust for geographic location [6] using the *gam *package [25] available in R v2.8.0 [26]. Two hypothesis tests were performed using the GAM framework: the CPT and FMSPT. When performing the CPT, an optimal span size for the observed data was selected through the application of GAM models to the observed data using a range of span sizes between 0.05 and 0.95. The AIC was recorded for each model and the span was selected to minimize the model AIC [6,12]. The span size was held constant as GAMs were applied to 999 permuted datasets. The statistic of interest was the difference in deviances between models including and excluding the smoothed term for geographic location. The null hypothesis was rejected if the observed difference in deviance statistic fell in the upper 2.5% of the distribution of statistics from permuted datasets [6,11].

The FMSPT was performed through the application of GAM models across either three or five predetermined span sizes, denoted by FMSPT-3 and FMSPT-5 respectively. The three span sizes selected were 0.1, 0.5, and 0.9 while the five span sizes were 0.1, 0.3, 0.5, 0.7, and 0.9. From each model the difference in deviance statistic was recorded and subsequently compared to the permutation distribution with the same span size. For FMSPT-3, the null hypothesis was rejected if the difference in deviance statistic fell in the upper 100*α3% of the distribution while for FMSPT-5, it was rejected if the statistic fell in the upper 100*α5% of the distribution [27].

Syntax used to generate data for this study and to minimize the AIC statistic across multiple span sizes using the software R v2.8.0 [26] is available on the Boston University Superfund Research Program website at: http://www.busbrp.org/projects/project2.html.

### Spatial Scan Statistic

The spatial scan statistic is a method that overlays the study region with overlapping circular regions centered at observation locations with radii varying continuously from zero to some specified upper limit (here, radii vary from zero to containing one-half of the study population). A *zone *is the infinite number of circles centered at some arbitrary point location with radii varying from zero to the upper bound. For a given radius, a zone can be further described by the number of individuals and cases falling inside the circle. The spatial scan statistic tests the null hypothesis of spatial randomness, i.e. the probability of disease within a circular zone equals that outside the zone, through a likelihood ratio test and detects the most likely cluster as the zone that maximizes the likelihood under the full parameter space. The distribution of the likelihood ratio depends on the underlying population distribution, upon which no assumptions have been made. With small samples it is possible to find the exact distribution; however for larger datasets Monte Carlo simulations are required [13].

In this study, the scan statistic was applied through the free available software SaTScan v 7.0.3 [17] using a purely spatial Bernoulli model, appropriate for case-control data. We rejected the null hypothesis if the most likely cluster was significant at the 0.05 level.

### Detecting Exposure Source Locations

We aimed to evaluate the ability of the GAM permutation tests and the spatial scan statistic to correctly identify the exposure source as high or low risk, i.e. the sensitivity of the methods. For Cases 1 and 2, we defined the CPT as successful in locating the exposure source if the global null hypothesis was rejected and the point-wise predicted logodds for the center of the study region fell in either the upper or lower 2.5% of the point-wise permutation distribution of predicted logodds. We considered the FMSPT successful if the global null hypothesis was rejected and at least one test predicted a point-wise logodds falling in the upper or lower 2.5% of the corresponding predicted permutation distribution. For Case 1, we also examined the proportion of the true cluster correctly identified as high or low risk by the point-wise tests, given that the global hypothesis was rejected. For Case 3, we defined sensitivity as the proportion of the vertical exposure source that was detected as increased risk for datasets when the global null hypothesis was rejected. For the FMSPTs, we present the proportion of the vertical source detected by at least one span size.

For Cases 1 and 2, if the center of the region was detected as part of a significant most likely cluster at the 0.05 level, the scan statistic was considered successful in identifying the area of risk. For Case 1, we also examined the percent of the true cluster overlapped by a significant most likely cluster. For Case 3, we examined the proportion of the vertical exposure source included in a significant most likely cluster.

The sensitivity to detect the exposure source is undefined for data simulated under the null hypothesis and so sensitivity estimates for these data are excluded. The local hypothesis tests are exploratory in nature but provide an additional measure by which the tests can be compared. We did not examine a measure of specificity in this analysis as, for Cases 2 and 3, the exposure source was not dichotomous in nature.

## Results

Theoretical powers for Cases 1, 2, and 3 were computed using equations from the literature [[28], 31, 32]. (See Additional file 1 for equations.) The theoretical power for a Pearson chi-square test applied to Case 1 ranged from 0.050 to greater than 0.980 for both probabilities of disease. For Case 2, the theoretical power ranged from 0.05 to 0.766 and 0.988 for probabilities of disease of 0.05 and 0.20, respectively. For Case 3, the power ranged from 0.05 to 0.935 and >0.999 for probabilities of disease of 0.05 and 0.20 at the edge of the region. (Table 2)

**Table 2:** Theoretical Power Based on Pearson Chi-Square Test and Simple Logistic Regression

	Probability of Disease Unexposed	Odds Ratios						
		0.5	1.0	1.5	2.0	2.5	3.0	3.5

Case 1^^^	0.05	0.258	0.050	0.214	0.598	0.884	>0.999	

	0.20	0.731	0.050	0.521	0.953	0.999	>0.999	

Case 2*	0.05	0.255	0.050	0.167	0.331	0.502	0.650	0.766

	0.20	0.565	0.050	0.321	0.663	0.872	0.959	0.988

Case 3*	0.05	0.311	0.050	0.215	0.464	0.695	0.850	0.935

	0.20	0.691	0.050	0.432	0.832	0.970	0.996	>0.999

^^^Power for comparison of odds inside to outside cluster

*Power for one standard deviation increase in distance from exposure source

The CPT outperformed FMSPT-3 and FMSPT-5 in each case. It was appropriately sized with observed type I error rates near 0.05. The power of the CPT was approximately doubled when comparing estimates for a probability of disease of 0.05 to 0.20 for each case. Larger power estimates were generally observed for Case 1, followed by Case 3 and Case 2. (Table 3)

**Table 3:** Observed Power for GAM Hypothesis Tests and Spatial Scan Statistic

Probability of Disease Unexposed		Odds Ratios						
		**0.5**	**1.0**	**1.5**	**2.0**	**2.5**	**3.0**	**3.5**

	**Case 1**	**Power** **(95% CI)**	**Power** **(95% CI)**	**Power** **(95% CI)**	**Power** **(95% CI)**	**Power** **(95% CI)**	**Power** **(95% CI)**	**Power** **(95% CI)**

0.05	CPT	0.081 (0.064-0.098)	0.043 (0.030-0.056)	0.070 (0.054-0.086)	0.174 (0.151-0.197)	0.301 (0.273-0.329)	0.474 (0.443-0.505)	
	FMSPT-3	0.055 (0.041-0.069)	0.033 (0.022-0.044)	0.048 (0.035-0.061)	0.139 (0.118-0.160)	0.250 (0.223-0.277)	0.403 (0.373-0.433)	
	FMSPT-5	0.044 (0.031-0.057)	0.023 (0.014-0.032)	0.039 (0.027-0.051)	0.103 (0.084-0.122)	0.225 (0.199-0.251)	0.361 (0.331-0.391)	
	Scan Statistic	0.087 (0.070-0.104)	0.052 (0.038-0.066)	0.075 (0.059-0.091)	0.169 (0.146-0.192)	0.302 (0.274-0.330)	0.494 (0.463-0.525)	

0.20	CPT	0.239 (0.213-0.265)	0.047 (0.034-0.06)	0.149 (0.127-0.171)	0.447 (0.416-0.478)	0.764 (0.738-0.79)	0.923 (0.906-0.94)	
	FMSPT-3	0.166 (0.143-0.189)	0.027 (0.017-0.037)	0.111 (0.092-0.130)	0.378 (0.348-0.408)	0.697 (0.669-0.725)	0.890 (0.871-0.909)	
	FMSPT-5	0.152 (0.130-0.174)	0.020 (0.011-0.029)	0.094 (0.076-0.112)	0.33 (0.301-0.359)	0.673 (0.644-0.702)	0.880 (0.860-0.900)	
	Scan Statistic	0.243 (0.216-0.270)	0.044 (0.031-0.057)	0.121 (0.101-0.141)	0.467 (0.436-0.498)	0.833 (0.810-0.856)	0.963 (0.951-0.975)	

	**Case 2**							

0.05	CPT	0.095 (0.077-0.113)	0.069 (0.053-0.085)	0.054 (0.040-0.068)	0.094 (0.076-0.112)	0.176 (0.152-0.200)	0.230 (0.204-0.256)	0.381 (0.351-0.411)
	FMSPT-3	0.074 (0.058-0.090)	0.048 (0.035-0.061)	0.035 (0.024-0.046)	0.075 (0.059-0.091)	0.131 (0.110-0.152)	0.186 (0.162-0.210)	0.31 (0.281-0.339)
	FMSPT-5	0.053 (0.039-0.067)	0.037 (0.025-0.049)	0.029 (0.019-0.039)	0.061 (0.046-0.076)	0.115 (0.095-0.135)	0.154 (0.132-0.176)	0.281 (0.253-0.309)
	Scan Statistic	0.059 (0.044-0.074)	0.052 (0.038-0.066)	0.048 (0.035-0.061)	0.091 (0.073-0.109)	0.137 (0.116-0.158)	0.194 (0.169-0.219)	0.293 (0.265-0.321)

0.20	CPT	0.223 (0.197-0.249)	0.05 (0.036-0.064)	0.118 (0.098-0.138)	0.293 (0.265-0.321)	0.520 (0.489-0.551)	0.714 (0.686-0.742)	0.878 (0.858-0.898)
	FMSPT-3	0.173 (0.150-0.196)	0.033 (0.022-0.044)	0.089 (0.071-0.107)	0.22 (0.194-0.246)	0.441 (0.410-0.472)	0.658 (0.629-0.687)	0.833 (0.810-0.856)
	FMSPT-5	0.147 (0.125-0.169)	0.023 (0.014-0.032)	0.073 (0.057-0.089)	0.199 (0.174-0.224)	0.404 (0.374-0.434)	0.611 (0.581-0.641)	0.798 (0.773-0.823)
	Scan Statistic	0.143 (0.121-0.165)	0.045 (0.032-0.058)	0.080 (0.063-0.097)	0.17 (0.147-0.193)	0.367 (0.337-0.397)	0.584 (0.553-0.615)	0.758 (0.731-0.785)

	**Case 3**							

0.05	CPT	0.094 (0.076-0.112)	0.040 (0.028-0.052)	0.059 (0.044-0.074)	0.131 (0.110-0.152)	0.240 (0.214-0.266)	0.426 (0.395-0.457)	0.547 (0.516-0.578)
	FMSPT-3	0.071 (0.055-0.087)	0.027 (0.017-0.037)	0.037 (0.025-0.049)	0.078 (0.061-0.095)	0.178 (0.154-0.202)	0.359 (0.329-0.389)	0.478 (0.447-0.509)
	FMSPT-5	0.056 (0.042-0.070)	0.017 (0.009-0.025)	0.028 (0.018-0.038)	0.064 (0.049-0.079)	0.152 (0.130-0.174)	0.324 (0.295-0.353)	0.437 (0.406-0.468)
	Scan Statistic	0.068 (0.052-0.084)	0.042 (0.030-0.054)	0.059 (0.044-0.074)	0.085 (0.068-0.102)	0.129 (0.108-0.15)	0.201 (0.176-0.226)	0.285 (0.257-0.313)

0.20	CPT	0.276 (0.248-0.304)	0.058 (0.044-0.072)	0.137 (0.116-0.158)	0.400 (0.370-0.430)	0.712 (0.684-0.740)	0.882 (0.862-0.902)	0.970 (0.959-0.981)
	FMSPT-3	0.218 (0.192-0.244)	0.036 (0.024-0.048)	0.094 (0.076-0.112)	0.327 (0.298-0.356)	0.622 (0.592-0.652)	0.843 (0.820-0.866)	0.954 (0.941-0.967)
	FMSPT-5	0.191 (0.167-0.215)	0.026 (0.016-0.036)	0.074 (0.058-0.090)	0.298 (0.270-0.326)	0.587 (0.556-0.618)	0.818 (0.794-0.842)	0.942 (0.928-0.956)
	Scan Statistic	0.158 (0.135-0.181)	0.047 (0.034-0.060)	0.067 (0.052-0.082)	0.192 (0.168-0.216)	0.348 (0.318-0.378)	0.534 (0.503-0.565)	0.703 (0.675-0.731)

In general, FMSPT-3 had higher power estimates than FMSPT-5, perhaps due to the slightly conservative Bonferroni-like significance cutoff adjustments. The greatest power estimates for the FMSPTs with an odds ratio of 3.0 were observed in Case 1 with a probability of disease for unexposed subjects of 0.20 and power estimates ranging from 0.027 (95%CI: 0.017-0.037) to 0.890 (95%CI: 0.871-0.909) and 0.020 (95%CI: 0.011-0.029) to 0.880 (95%CI: 0.860-0.900) for the 3 and 5 span tests, respectively. The power estimates for Case 3 were greater than those of Case 2 with maximal power estimates of 0.954 (95%CI: 0.941-0.967) and 0.942 (95%CI: 0.928-0.956) observed for FMSPT-3 and FMSPT-5, respectively, for an odds ratio of 3.5 in Case 3 and 0.833 - (95%CI: 0.810-0.856) and 0.798 (95%CI: 0.773-0.823) in Case 2. (Table 3)

The spatial scan statistic performed best in Case 1, followed by Cases 2 and 3. In Case 1, the scan statistic had a maximum estimated power of 0.963 (95%CI: 0.951-0.975) with a probability of disease outside the cluster of 0.20. In Cases 2 and 3 the maximum power was 0.758 (95%CI: 0.731-0.785) and 0.703 (95%CI: 0.675-0.731), respectively, for an odds ratio of 3.5. The test had an appropriate type I error rate for all cases and scenarios. It was outperformed by all three of the permutation testing methods in Cases 2 and 3 but had the highest power estimates in Case 1. (Table 3)

Examining method sensitivity, in Case 1, the CPT detected an average proportion of 0.740 (SD: 0.202) and 0.855 (SD: 0.140) of the true cluster as a hotspot with an odds ratio of 3.0 and probabilities of disease outside the cluster of 0.05 and 0.20, respectively. For the same odds ratio and scenarios, FMSPT-3 outperformed the CPT detecting 0.855 (SD: 0.150) and 0.961 (SD: 0.063), while FMSPT-5 detected 0.876 (SD: 0.133) and 0.969 (SD: 0.058). The scan statistic had the smallest average proportion of the true cluster detected with averages of 0.686 (SD: 0.290) and 0.842 (SD: 0.186) for an odds ratio of 3.0 and probabilities of disease outside the cluster of 0.05 and 0.20, respectively. (Table 4)

**Table 4:** Case 1 Sensitivity - Mean Proportion of True Cluster Detected as Hot- or Coldspot

		Odds Ratios				
		**0.5**	**1.5**	**2.0**	**2.5**	**3.0**
**Probability of Disease Unexposed**		**Mean** **(SD)**	**Mean** **(SD)**	**Mean** **(SD)**	**Mean** **(SD)**	**Mean** **(SD)**

0.05	CPT	0.380 (0.287)	0.360 (0.240)	0.582 (0.258)	0.671 (0.221)	0.740 (0.202)
	FMSPT-3	0.418 (0.493)	0.513 (0.256)	0.697 (0.259)	0.807 (0.179)	0.855 (0.150)
	FMSPT-5	0.364 (0.481)	0.538 (0.249)	0.725 (0.261)	0.834 (0.169)	0.876 (0.133)
	Scan Statistic	0.335 (0.435)	0.325 (0.331)	0.536 (0.359)	0.607 (0.320)	0.686 (0.290)

0.20	CPT	0.653 (0.251)	0.516 (0.278)	0.738 (0.218)	0.811 (0.172)	0.855 (0.140)
	FMSPT-3	0.584 (0.493)	0.644 (0.288)	0.851 (0.156)	0.926 (0.104)	0.961 (0.063)
	FMSPT-5	0.618 (0.486)	0.660 (0.292)	0.874 (0.141)	0.935 (0.098)	0.969 (0.058)
	Scan Statistic	0.684 (0.333)	0.498 (0.380)	0.688 (0.303)	0.782 (0.242)	0.842 (0.186)

*Senstivity = mean(Percent Cluster Located | Rejected Ho)

Examining the ability of the methods to detect the exposure source location, the CPT was outperformed by both FMSPTs in Case 2. When the null hypothesis was rejected, the exposure source was correctly identified as a point of high or low risk by the CPT in up to 98.7% (95%CI: 98.0-99.4%) of datasets. (Table 5) The estimates for the FMSPTs were greater, with the exposure source correctly identified in over 99% of datasets where the global null hypothesis was rejected with an odds ratio of 3.5 and a probability of disease for unexposed subjects of 0.20. (Table 5) The scan statistic was outperformed by the permutation testing methods in Case 2. It detected the exposure source in 72.5% (95%CI: 69.7-75.3%) of datasets where the null hypothesis was rejected with a probability of disease for unexposed subjects of 0.20 and an odds ratio of 3.5. (Table 5)

**Table 5:** Cases 2 and 3 Sensitivity - Detecting the Exposure Source Location

Probability of Disease Unexposed		Odds Ratios					
		**0.5**	**1.5**	**2.0**	**2.5**	**3.0**	**3.5**

	**Case 2**^**^**^	**Sensitivity** **(95% CI)**	**Sensitivity** **(95% CI)**	**Sensitivity** **(95% CI)**	**Sensitivity** **(95% CI)**	**Sensitivity** **(95% CI)**	**Sensitivity** **(95% CI)**

0.05	CPT	0.453 (0.422-0.484)	0.352 (0.322-0.382)	0.543 (0.512-0.574)	0.688 (0.659-0.717)	0.896 (0.877-0.915)	0.882 (0.862-0.902)
	FMSPT-3	0.541 (0.510-0.572)	0.543 (0.512-0.574)	0.773 (0.747-0.799)	0.824 (0.800-0.848)	0.952 (0.939-0.965)	0.952 (0.939-0.965)
	FMSPT-5	0.585 (0.554-0.616)	0.517 (0.486-0.548)	0.754 (0.727-0.781)	0.8 (0.775-0.825)	0.968 (0.957-0.979)	0.947 (0.933-0.961)
	Scan Statistic	0.026 (0.016-0.036)	0.012 (0.005-0.019)	0.038 (0.026-0.050)	0.094 (0.076-0.112)	0.153 (0.131-0.175)	0.229 (0.203-0.255)

0.20	CPT	0.740 (0.713-0.767)	0.559 (0.528-0.590)	0.860 (0.838-0.882)	0.950 (0.936-0.964)	0.976 (0.967-0.985)	0.987 (0.980-0.994)
	FMSPT-3	0.936 (0.921-0.951)	0.652 (0.622-0.682)	0.964 (0.952-0.976)	0.995 (0.991-0.999)	0.995 (0.991-0.999)	0.998 (0.995-1.000)
	FMSPT-5	0.973 (0.963-0.983)	0.644 (0.614-0.674)	0.965 (0.954-0.976)	0.995 (0.991-0.999)	0.997 (0.994-1.000)	0.999 (0.997-1.000)
	Scan Statistic	0.082 (0.065-0.099)	0.029 (0.019-0.039)	0.12 (0.100-0.140)	0.301 (0.273-0.329)	0.533 (0.502-0.564)	0.725 (0.697-0.753)

	Case 3*	Mean (SD)	Mean (SD)	Mean (SD)	Mean (SD)	Mean (SD)	Mean (SD)

0.05	CPT	0.256 (0.097)	0.234 (0.069)	0.333 (0.134)	0.391 (0.190)	0.463 (0.258)	0.524 (0.293)
	FMSPT-3	0.308 (0.101)	0.344 (0.072)	0.436 (0.129)	0.516 (0.213)	0.59 (0.301)	0.634 (0.334)
	FMSPT-5	0.368 (0.101)	0.353 (0.065)	0.473 (0.126)	0.549 (0.209)	0.622 (0.306)	0.661 (0.343)
	Scan Statistic	0.263 (0.319)	0.189 (0.234)	0.232 (0.261)	0.299 (0.279)	0.315 (0.268)	0.346 (0.277)

0.20	CPT	0.394 (0.203)	0.339 (0.134)	0.454 (0.253)	0.548 (0.286)	0.641 (0.255)	0.704 (0.192)
	FMSPT-3	0.511 (0.228)	0.453 (0.145)	0.574 (0.287)	0.659 (0.340)	0.737 (0.296)	0.788 (0.204)
	FMSPT-5	0.550 (0.229)	0.475 (0.136)	0.598 (0.290)	0.638 (0.355)	0.758 (0.317)	0.807 (0.222)
	Scan Statistic	0.363 (0.280)	0.242 (0.280)	0.282 (0.300)	0.390 (0.287)	0.441 (0.267)	0.443 (0.257)

^^^Sensitivity reflects the probability of the identification of the center of the region as high or low risk, given that the global null hypothesis was rejected, i.e.

Sensitivity = P(Exposure Source Located | Rejected Ho)

* Sensitivity reflects the mean percent of the vertical exposure source identified as high or low risk, given that the global null hypothesis was rejected, i.e.

Sensitivity = mean(Percent Exposure Source Located | Rejected Ho)

In Case 3, the FMSPTs outperformed the CPT and scan statistic detecting a higher proportion of the vertical exposure source. For probabilities of disease of 0.05 and 0.20, FMSPT-3 detected an average proportion of 0.634 (SD: 0.334) and 0.788 (SD: 0.204) and FMSPT-5 detected 0.661 (SD: 0.343) and 0.807 (SD: 0.222) of the vertical exposure source, respectively, for an odds ratio of 3.5. The CPT detected proportions of 0.524 (SD: 0.293) and 0.704 (SD: 0.192) on average while the scan statistic had mean detection proportions of 0.346 (SD: 0.277) and 0.443 (SD: 0.257) for the probabilities of disease 0.05 and 0.20, respectively. (Table 5)

**Figure 5:**
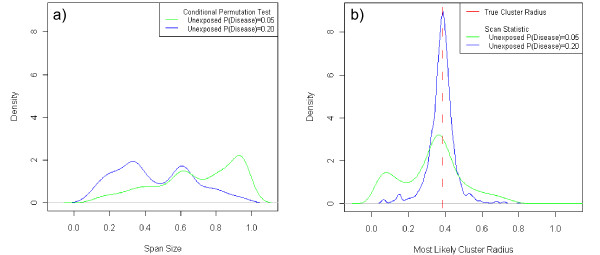
**Distributions of Optimal Span Size and Most Likely Cluster Radius Observed for Case 1 with Odds Ratio of 3.0.** a: Case 1 Conditional Permutation Test Optimal Span Size for Odds Ratio of 3.0. This figure depicts the optimal span size selected by applying GAMs across a range of possible spans and selecting the optimal span as that which corresponds to the minimal model AIC statistic. b: Case 1 Scan Statistic Most Likely Cluster Radius for Odds Ratio of 3.0. This figure depicts the distribution of the observed radius for most likely clusters selected by the scan statistic. It is paired with Figure 4a as we can compare the tendencies of the methods to over- or under-smooth through these figures. With Figure 4a we see that for lower disease prevalence the GAM methods tend to choose a large span size, possibly over-smoothing and missing the cluster. The scan statistic tends to under-smooth and finds a most likely cluster that is much smaller than the true cluster radius, as shown in Figure 4b.

The distribution of the selected span size for the CPT was bimodal with models near 0.3 and 0.6 for Case 1 for a probability of disease outside the cluster of 0.20. For a probability of disease of 0.05, there was greater density of the distribution near large span sizes indicating that for smaller probabilities of disease for unexposed subjects, the GAM was more likely to choose large span sizes than for higher prevalence diseases. (Figure 5a) The distribution of the radius of the scan statistic for Case 1, for a probability of disease for unexposed subjects of 0.20 and an odds ratio of 3.0, was unimodal with both mean and mode near the true cluster radius of $\sqrt{0.15}$. (Table 6) The distribution for a probability of disease for unexposed subjects of 0.05 was bimodal with modes near 0.1 and the true cluster size showing a tendency of the scan statistic to detect small clusters when analyzing diseases of lower prevalence. (Figure 5b)

**Table 6:** Radii of Scan Statistic Most Likely Cluster with Significant P-Value (p < 0.05)

	Odds Ratios						
	
	0.5	1.0	1.5	2.0	2.5	3.0	3.5
	**Mean** **(SD;Min-Max)**	**Mean** **(SD;Min-Max)**	**Mean** **(SD;Min-Max)**	**Mean** **(SD;Min-Max)**	**Mean** **(SD;Min-Max)**	**Mean** **(SD;Min-Max)**	**Mean** **(SD;Min-Max)**

Case 1							

0.05	0.39 (0.25;0.04-1.12)	0.34 (0.26;0.04-0.95)	0.32 (0.24;0.03-1.09)	0.37 (0.19;0.03-0.91)	0.35 (0.16;0.03-1.02)	0.37 (0.14;0.02-0.91)	

0.20	0.37 (0.14;0.05-0.88)	0.24 (0.13;0.08-0.68)	0.33 (0.19;0.04-0.95)	0.37 (0.13;0.06-1.03)	0.38 (0.09;0.07-0.71)	0.38 (0.07;0.07-0.80)	

Case 2							

0.05	0.37 (0.23;0.04-0.70)	0.33 (0.24;0.05-0.96)	0.31 (0.22;0.04-1.01)	0.36 (0.26;0.03-1.10)	0.43 (0.24;0.04-1.09)	0.43 (0.2;0.04-0.84)	0.45 (0.19;0.04-1.00)

0.20	0.4 (0.21;0.04-1.12)	0.28 (0.18;0.07-0.82)	0.36 (0.21;0.07-0.77)	0.45 (0.19;0.07-0.84)	0.47 (0.18;0.06-0.71)	0.50 (0.16;0.07-0.92)	0.53 (0.14;0.06-0.73)

Case 3							

0.05	0.4 (0.29;0.04-0.95)	0.43 (0.32;0.06-1.06)	0.34 (0.26;0.05-1.03)	0.35 (0.24;0.06-0.94)	0.41 (0.24;0.04-0.85)	0.47 (0.22;0.05-1.02)	0.46 (0.22;0.04-1.10)

0.20	0.46 (0.22;0.06-1.1)	0.38 (0.22;0.09-0.88)	0.39 (0.24;0.08-1.36)	0.41 (0.24;0.06-1.13)	0.49 (0.22;0.06-1.05)	0.52 (0.20;0.07-0.89)	0.52 (0.19;0.07-0.92)

**Figure 6:**
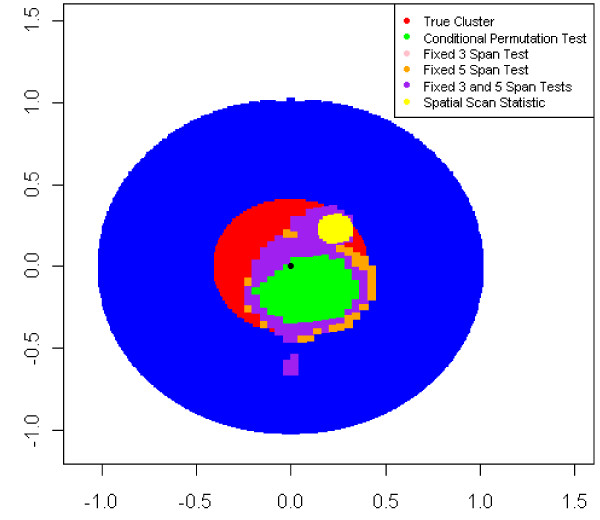
**Case 1 Points Detected at High Risk for Data with Scan Statistic Minimum Radius, Probability of Disease Outside Cluster = 0.20**. This figure compares the area of the region detected as high risk by the methods discussed in this paper. This particular figure shows the minimum radius observed for a significant most likely cluster with an odds ratio of 3.0 and a probability of disease outside the cluster of 0.20.

**Figure 7:**
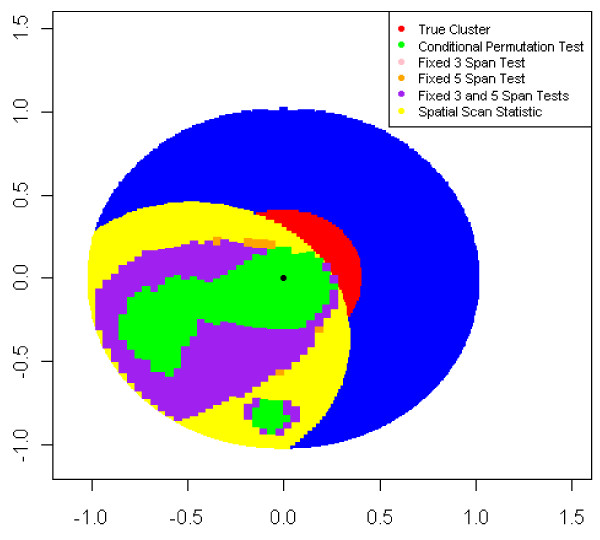
**Case 1 Points Detected at High Risk for Data with Scan Statistic Maximum Radius, Probability of Disease Outside Cluster = 0.20**. This figure compares the area of the region detected as high risk by the methods discussed in this paper. This particular figure shows the maximum radius observed for a significant most likely cluster with an odds ratio of 3.0 and a probability of disease outside the cluster of 0.20.

Figures 6 and 7 display two datasets from Case 1 with an odds ratio of 3.0 and a probability of disease of 0.20 for unexposed subjects where the global null hypothesis was rejected by all of the methods compared. In Figure 6, the CPT detected between 6.3 and 33.7% of points as increased risk while FMSPT-3 and FMSPT-5 detected 12.8-38.1% and 13.7-38.2% of points, respectively. For the same case, odds ratio, and probability of disease, the scan statistic detected a cluster with the smallest radius observed, 0.07, while in Figure 7 it detected its largest cluster with a radius of 0.80.

## Discussion

Simulated data were used to compare the power and sensitivity of the CPT and FMSPTs performed with GAMs to the spatial scan statistic under three simple alternative hypotheses. Theoretical power was computed for each alternative hypothesis to provide a comparison of spatial statistic hypothesis tests to simpler methods.

In Case 1, a circular cluster was centered in the study region. The spatial scan statistic identifies clusters by placing circular zones across the region of interest and comparing the likelihood of disease within to outside the zones. As this method is similar to the pattern of disease risk for this Case, it is unsurprising that the scan statistic had the highest estimated power, nearing the theoretical power calculated for a Pearson chi-square test. The CPT had slightly lower power than the scan statistic and FMSPT-3 and FMSPT-5 had lower power estimates.

In Case 2, there was a linear association between Euclidean distance from the center of the circular study region and the logodds of disease. Case 3 was a square study region with a linear association between the proximity to the center of the horizontal axis and the logodds of disease. As these cases would be appropriately analyzed by logistic regression methods, GAM permutation tests had an advantage over the scan statistic in its flexibility to detect different patterns in disease risk. In both Cases 2 and 3, the CPT had the highest estimated power though estimates were at least 10% smaller than the theoretical power of a logistic regression. The scan statistic had the lowest power for Cases 2 and 3. For all tests, power estimates for Case 1 exceeded those of Cases 2 and 3. For the GAM permutation tests, power estimates for Case 3 were greater than those of Case 2 under similar conditions while the estimates were comparable between the two cases for the scan statistic.

The size of the most likely cluster and hot- and coldspots identified by the scan statistic and GAM methods varied greatly across datasets, as observed in Figures 6 and 7. For Case 1, an odds ratio of 3.0, and a lower prevalence, i.e. a probability of disease outside the cluster of 0.05, the spatial scan statistic had larger variation in most likely cluster radius and a greater probability of a most likely cluster having a radius smaller than the true cluster than for higher prevalence. (Table 6, Figure 5a) The scan statistic showed a tendency to detect small clusters while the CPT tended to smooth over small variations in disease risk as a large span (span > 0.80) was more likely to be selected when analyzing diseases of lower prevalence. (Figure 5b)

Comparing model sensitivities, in Case 1, the FMSPT-5 consistently detected the highest proportion of the true cluster as a hot- or coldspot, followed by FMSPT-3 and CPT with the scan statistic having the lowest mean proportion detected. It is not surprising that FMSPT-3 and FMSPT-5 had the highest sensitivity estimates as the definition of sensitivity of these tests considered points detected if they were considered a hot- or coldspot in at least one of 3 or 5 models. Sensitivity for the CPT required the points to be detected at a single span size.

Of interest, the spatial scan statistic had the highest power estimates for Case 1 though it did not detect the highest proportion of the true cluster. As for its sensitivity, the scan statistic detected a most likely cluster of the correct size with a radius within ±0.01 of the true cluster radius in 19.4% of datasets with an odds ratio of 3.0 and a probability of disease outside the cluster of 0.20. Of these most likely clusters, 12.4% were centered in the correct location and only one dataset was observed to have a correct cluster radius and location with a p-value of less than 0.05.

In Case 2, sensitivity was measured by the probability of detecting the exposure source, given that the global null hypothesis was rejected. In practice, after detecting variation in disease risk, public health resources may be sent to specific locations detected as hot- or coldspots to determine the source of exposure. If the exposure point source is not included in the most likely cluster or hot-/coldspot detected, it is unlikely that public health officials will be able identify the true exposure that is increasing disease risk. A minimum sensitivity of 80% may be considered a reasonable requirement of tests used for application. The FMSPTs had sensitivity estimates of at least 80% for odds ratios over 2.0 while the CPT had sensitivities of 80% for odds ratios of at least 3.0 for both probabilities of disease. The sensitivity of the scan statistic did not reach 80% for any odds ratios, having much lower estimates than the permutation testing methods. Of the datasets where the scan statistic detected a most likely cluster with a p-value of less than 0.05, it rarely identified the correct exposure point source.

Sensitivity for Case 3 was measured as the proportion of the vertical exposure source identified as high or low risk, given that the global null hypothesis was rejected. Again, the spatial scan statistic had much lower sensitivity than the permutation testing methods. For odds ratios of at least 3.0 and a probability of disease for unexposed subjects of 0.20, the FMSPTs had sensitivity estimates of at least 70%, slightly lower than the desired magnitude. FMSPT-5 had the highest sensitivity, followed by FMSPT-3 and CPT.

For the CPT, we selected the span size through minimization of the AIC statistic. Many other methods of span selection are available. We believe similar results would be observed for any data driven span selection procedure, but further research is needed to confirm this. For the FMSPTs, we selected spans for a range across possible span sizes *a priori*. Other span sizes could be selected and power estimates may change accordingly. For the CPT and FMSPTs, we applied significance level adjustments based on empirical evidence from previous research and a nominal α level of 0.05 [27]. There is no guarantee that similar results will be observed in future studies as the significance cutoffs used here were selected and evaluated through a single set of simulations. For different nominal α levels, appropriate significance cutoffs must be determined. A number of extensions to the scan statistic are available, including elliptical [28] and flexibly shaped [29] zones; however for this research, our interest was in evaluating the original, and widely used, circular spatial scan statistic as applied using the software SaTScan. Applications of other versions of the scan statistic may influence the statistical power and sensitivity of the test. Evaluation of the extended methods is left for future research. In this research, we applied the methods to point data. Both the scan statistic and GAM methods are applicable to aggregate data and if applied to such data, the resulting distribution of power estimates would likely change.

## Conclusions

Power of at least 80% indicates that the null hypothesis is correctly rejected at a high rate, a desirable quality of a testing method. The permutation tests each had power estimates exceeding the 80% threshold for large odds ratios. Reduced power was observed for a lower prevalence disease, as was expected with reduced theoretical power. The scan statistic had an observed power estimate of at least 80% for a circular cluster of increased risk centered in the study region but lower estimates for other variations in disease risk.

Sensitivities of at least 80% are desirable to ensure that the testing methods detect the correct areas of increased or decreased risk. In general, the FMSPTs had the highest sensitivity with estimates of at least 80% with large odds ratios for all disease risk patterns examined. The CPT had slightly lower sensitivity though its sensitivity reached 80% for higher prevalence diseases and with large odds ratios. The scan statistic had lower sensitivity estimates for all variations of disease risk examined and was observed to have at least 80% sensitivity only for a circular cluster centered in the study region which mimicked its own cluster detection method.

Simple patterns of spatial variation in disease risk were considered in this study. The relative pattern of power estimates of the four methods differ based on the pattern of disease risk considered. The spatial scan statistic outperformed the GAM methods in the case of a circular cluster centered in the study region, though it underperformed in sensitivity. For a linear association between geographic location and disease risk, the scan statistic had power estimates and sensitivity falling below the GAM estimates. It is important to note that analyses were performed using point data. Results of power comparisons applied to aggregate data may differ from those observed here. Across all simple scenarios examined in this research, the GAM methods presented a reasonable alternative with similar or greater power estimates and sensitivity exceeding that of the spatial scan statistic.

## Competing interests

The authors declare that they have no competing interests.

## Authors' contributions

RLY designed and performed simulation studies, drafted and revised the manuscript, and has approved the final version for submission to this journal. JW, VV, AO, and TFW participated in study design, made significant revisions and contributions to the manuscript, and approved the final version for submission to this journal.

## Supplementary Material

Additional file 1 **Theoretical Power**. This file includes information regarding how to compute the theoretical power for the Pearson chi-square test and logistic regressions applied. Click here for file
