# Supplementary material for: A power comparison of generalized additive models and the spatial scan statistic in a case-control setting
Source: Int J Health Geogr. 2010 Jul 19;9:37. doi: 10.1186/1476-072X-9-37 (PMC2918545; doi:10.1186/1476-072X-9-37)
Supplement: Additional file 1 — Theoretical Power. This file includes information regarding how to compute the theoretical power for the Pearson chi-square test and logistic regressions applied. [file 1476-072X-9-37-S1.DOC]

**Additional file 1**

**Theoretical Power**

Data for Case 1 could be analyzed using a Pearson chi-square test. To provide a theoretical comparison of the power of the complex spatial hypothesis tests we computed the theoretical power of a Pearson chi-square test:

$$Power=P\left(\chi_{ncp=nw^{2},df=1}^{2}\geq\chi_{ncp=1,df=1,\alpha=0.05}^{2}\right)$$

Where *ncp* is the noncentrality parameter equal to the sample size multiplied by the effect size, *w,* squared with

$w=\sqrt{\sum_{i=1}^{2} \frac{\left(p_{0i}-p_{1i}\right)^{2}}{p_{0i}}}$ .

Here, $p_{01}$ and $p_{02}$ are the joint-probabilities of controls and $p_{11}$ and $p_{12}$ represent the joint-probabilities of cases living inside and outside the cluster, respectively. [1]

Data for Cases 2 and 3 could be appropriately analyzed using a logistic regression. To evaluate the performance of the spatial hypothesis tests, we computed the theoretical power of detecting an association between the occurrence of disease and a one standard deviation increase in distance from the exposure source, i.e. distance from the center of the study region for Case 2 and from the center of the horizontal axis for Case 3 (approximately 23 and 28% of the distance for Cases 2 and 3, respectively).

$Power=P\left(Z\geq z_{\beta}\right)$,

with

,

where

$$\alpha=1-P\left(Z>z_{\alpha}\right)$$

$\delta=\frac{1+\left(1+\lambda^{2}\right)\exp\left(\frac{5\lambda^{2}}{4}\right)}{1+\exp\left(\frac{-\lambda^{2}}{4}\right)}$.

$\bar{\pi}$ is the probability of a case at the mean distance from the center of the region. $\lambda$ is the logodds for a distance that is one standard deviation further than the mean distance from the center of the region. [2, 3]

**References**

1. Cohen J: *Statistical power analysis for the behavioral sciences.* 2nd edn. Hillsdale, NJ: Earlbaum; 1988.

2. Agresti A: *An Introduction to Categorical Data Analysis.* New York: A Wiley-Interscience Publication; 1996.

3. Hsieh FY: **Sample size tables for logistic regression.** *Statistics in Medicine* 1989, **8:**795-802.
